# Effect of Culture-Independent Diagnostic Tests on Future Emerging Infections Program Surveillance

**DOI:** 10.3201/eid2109.150570

**Published:** 2015-09

**Authors:** Gayle Langley, John Besser, Martha Iwamoto, Fernanda C. Lessa, Alicia Cronquist, Tami H. Skoff, Sandra Chaves, Dave Boxrud, Robert W. Pinner, Lee H. Harrison

**Affiliations:** Centers for Disease Control and Prevention, Atlanta, Georgia, USA (G. Langley, J. Besser, M. Iwamoto, F.C. Lessa, T.H. Skoff, S. Chaves, R.W. Pinner);; Colorado Department of Public Health and Environment, Denver, Colorado, USA (A. Cronquist);; Minnesota Department of Health, St. Paul, Minnesota, USA (D. Boxrud);; Johns Hopkins Bloomberg School of Public Health, Baltimore, Maryland, USA (L.H. Harrison)

**Keywords:** culture-independent diagnostics, surveillance, molecular testing, antigen-based testing, Emerging Infections Program, EIP

## Abstract

The Centers for Disease Control and Prevention Emerging Infections Program (EIP) network conducts population-based surveillance for pathogens of public health importance. Central to obtaining estimates of disease burden and tracking microbiological characteristics of these infections is accurate laboratory detection of pathogens. The use of culture-independent diagnostic tests (CIDTs) in clinical settings presents both opportunities and challenges to EIP surveillance. Because CIDTs offer better sensitivity than culture and are relatively easy to perform, their use could potentially improve estimates of disease burden. However, changes in clinical testing practices, use of tests with different sensitivities and specificities, and changes to case definitions make it challenging to monitor trends. Isolates are still needed for performing strain typing, antimicrobial resistance testing, and identifying other molecular characteristics of organisms. In this article, we outline current and future EIP activities to address issues associated with adoption of CIDTs, which may apply to other public health surveillance.

## Abstract 2

Use of such tests in clinical settings presents opportunities and challenges.

The Centers for Disease Control and Prevention (CDC) Emerging Infections Program (EIP) network conducts population- and laboratory-based surveillance for foodborne, health care–associated, respiratory, and invasive bacterial pathogens of public health importance. The main objectives of surveillance are to 1) measure disease burden and monitor disease trends over time, 2) evaluate the impact of public health interventions, 3) track microbiological and molecular characteristics of pathogens, and 4) detect emerging infectious disease threats. EIP data are used for national projections of disease incidence and formulation of national public health policy for prevention and control of disease. Central to accomplishing these objectives is accurate laboratory detection of the pathogens under surveillance.

In the field of microbiology, culture remains the standard for detection of most organisms, but in clinical settings, detection of pathogens is increasingly reliant on culture-independent diagnostic tests (CIDTs). CIDTs include antigen-based tests and molecular tests. The most commonly used molecular tests are the nucleic acid amplification tests, which include PCR. In clinical settings, most CIDTs have several advantages over culture. Foremost, CIDT results can be obtained more rapidly than culture, a feature that can be critical for clinical decision-making. Additionally, CIDTs may require less technical expertise to perform. Although initial adoption of these newer technologies can be expensive, costs generally decline over time, particularly those associated with labor.

CIDTs have the potential to improve estimates of disease burden because 1) they may be more sensitive than culture, 2) their relative ease of use may increase the number of patients tested, 3) they may enable detection of organisms for which there are currently no practical laboratory tests, and 4) they may increase the ability to detect polymicrobial infections. However, incorporating CIDTs into public health surveillance presents several challenges. Interpreting trends in disease incidence can be difficult because of changes to testing practices and surveillance case definitions. Although also true for culture, detection of molecular material may not reflect the presence of a living microbe and true disease, especially when detected from nonsterile body sites. At least for now, it is generally more difficult to assess microbiological and molecular characteristics, such as pathogen subtypes and antimicrobial drug resistance and genotypes, without bacterial isolates. Addressing these and other factors that affect estimates of disease burden and the characterization of infectious pathogens is critical for public health surveillance systems and clinical decision-making. EIP sites have a long history of close collaboration between CDC, state and local public health departments, academia, and clinical laboratories, making them uniquely positioned to help chart the course in addressing these concerns. Because many infections are already being diagnosed by use of CIDTs and more CIDTs will probably be developed and used in the near future, a path for addressing these issues is urgently needed. This article provides an overview of current testing practices for pathogens under EIP surveillance and addresses how EIPs plan to advance their core objectives in the face of this dynamic diagnostic environment.

## Current Status of CIDTs in the EIP Network

CIDTs are either singleplex (i.e., they test for a single organism) or multiplex (i.e., they simultaneously test for multiple organisms). There has been rapid development of multiplex molecular tests that detect pathogens commonly associated with particular syndromes (e.g., respiratory, enteric, and bloodstream infections). CIDTs can be classified into commercial test kits that receive Food and Drug Administration (FDA) clearance or laboratory-developed tests (LDTs). FDA-cleared CIDTs undergo various levels of validation before they are made available for purchase in the United States, but postmarketing evaluations are generally not required (http://www.fda.gov/MedicalDevices/ProductsandMedicalProcedures/InVitroDiagnostics/ucm407296.htm). FDA defines LDTs as “in vitro diagnostic tests that are designed, manufactured, and used within a single laboratory.” Laboratories are required to establish test characteristics for LDTs, including accuracy and precision. Historically, FDA has not generally enforced premarket review and other applicable requirements because LDTs were relatively simple and available on a limited basis. Many LDTs are now more complex and are used nationwide. FDA guidance on additional oversight of LDTs is pending. (http://www.fda.gov/MedicalDevices/ProductsandMedicalProcedures/InVitroDiagnostics/ucm407296.htm).

Many EIPs regularly conduct systematic surveys of clinical, commercial, and public health laboratories to monitor the use of CIDTs in laboratories that provide services to the population under surveillance. These surveys show that the availability and type of CIDTs used varies by pathogen (Table 1). All or nearly all cases of influenza, *Clostridium difficile, Legionella* spp., and *Bordetella pertussis* infection reported through EIP are diagnosed by CIDTs. The percentage diagnosed by a particular type of CIDT has varied over the years. For instance, rapid antigen tests for influenza have been increasingly replaced by FDA-cleared molecular assays (*1*), including multiplex assays to detect viruses and bacteria from respiratory specimens (*2*)*.* Molecular tests are increasingly being used to detect *C. difficile* infection. During 2011, ≈50% of *C. difficile* infections were diagnosed by molecular assays performed at laboratories that serve the EIP population (*3*)*.* Also in 2011, for surveillance of *Legionella* infections, 95% of cases were diagnosed by detection of urine antigen for *L. pneumophila* serogroup 1 (http://www.cdc.gov/abcs/reports-findings/survreports/leg12.pdf). During the early 1990s, culture and direct fluorescent antibody testing were the primary diagnostic methods used to identify *B. pertussis* cases reported through the National Notifiable Disease Surveillance System (*4*). PCR, either alone or in combination with other diagnostic tests, diagnosed 89% of laboratory-confirmed pertussis cases reported through the EIP Enhanced Pertussis Surveillance system during 2011–2014 (CDC, unpub. data).

**Table 1:** Case definitions and status of microbiological testing, by Emerging Infections Program activity and pathogen, 2010–2014*

Program, pathogen (year surveillance started)	Case definition criteria	Cases identified by culture, %	FDA-approved CIDTs related to EIP case definitions, by test type/% laboratories offering test (year data collected)†	Laboratories offering laboratory-developed NATs (year data collected), %†	Additional cases, %‡
Active Bacterial Core surveillance					
*Streptococcus pneumoniae* (1995)	Isolation from sterile site	100	ABT on sterile site specimens/13 (2012)	1% (2014)	Not available
*Haemophilus influenzae* (1995)	Isolation from sterile site	100	ABT on sterile site specimens/7 (2012)	<1 (2014)	Not available
*Neisseria meningitidis* (1995)	Isolation from sterile site	100	ABT on sterile sites specimens/8 (2012)	1 (2014)	9§
Group A *Streptococcus* (1995)	Isolation from sterile site	100	None on direct specimen from sterile site	1 (2014)	Not available
Group B *Streptococcus*(1995)	Isolation from sterile site	100	None on direct specimen from sterile site	1 (2014)	Not available
Methicillin-resistant *Staphylococcus aureus* (2005)	Isolation from sterile site	100	None on direct specimen from sterile site	1 (2014)	Not available
Pertussis (2011)	Clinical criteria plus isolation from clinical specimen or PCR positive	<1	None	Not available	Not available
Legionellosis (2011)	Isolation, positive urine antigen or rise in serologic titers¶	5	Urine antigen test/92 (2010–2011)#	47.5 (2010–2011)#	<1
FoodNet					
* Campylobacter*	Isolation from clinical specimen	100	ABT/14 (2014) NAT multiplex/1 (2014)	1 (2014)	13
* Salmonella*	Isolation from clinical specimen	100	NAT multiplex/1 (2014)	1 (2014)	1.3
*Shigella*	Isolation from clinical specimen	100	NAT multiplex/0.5 (2014)	0.5 (2014)	5
*Vibrio*	Isolation from clinical specimen	100	NAT multiplex/0 (2014)	0.6 (2014)	2
*Yersinia*	Isolation from clinical specimen	100	NAT multiplex/0 (2014)	0.5 (2014)	2
*Listeria*	Isolation from sterile site	100	NAT-based multiplex/0 (2014)	0 (2014)	0
* Cryptosporidium*	Detection of organism, antigen, or nucleic acid	0	ABT/88 (2014); NAT multiplex/1 (2014)	2 (2014)	Not applicable
*Cyclospora*	Detection of organism or nucleic acid	0	NAT multiplex/0 (2014)	0	Not applicable
Shiga toxin–producing *Escherichia coli*	Isolation of *E. coli* O157:H7; or, isolation of *E. coli* with detection of Shiga toxin or genes that encode the toxins	100	ABT/57 (2014); NAT multiplex/1 (2014)	1	8
Health care–associated infections					
Gram-negative bacilli *E. coli, Klebsiella pneumoniae, K. oxytoca, Enterobacter cloacae, Enterobacter aerogenes, Acinetobacter baumannii*	Isolation from a sterile site or urine determined to be not susceptible to certain antimicrobial organisms**	100	None on direct specimen from sterile site	Not available	Not available
*Candida* spp.	Isolation from blood	100	Magnetic resonance test for multiple species/offering unknown	Not available	Not available
* Clostridium difficile*	Positive toxin or molecular assay from stool specimen	0	NAT for *C. difficile*/58 (2013) NAT multiplex/1 (2013) toxin-based ABT/37 (2013)	2 (2013)	Not applicable
Influenza-associated hospitalizations					
Influenza virus	Isolation or detection from fluorescent antibody staining, antigen test or RT-PCR	<2††	ABTs NAT (singleplex or multiplex)/24 (2013)‡‡	2	Not applicable

*Data from EIP laboratory surveys. ABT, antigen-based test; CIDT, culture-independent diagnostic test; EIP, Emerging Infections Program; FDA, Food and Drug Administration; NAT, nucleic acid test; RT-PCR, reverse transcription PCR. †Laboratory tests may be offered either onsite or offsite. ‡Estimated additional cases that could be captured by CIDTs in use. These infections are not counted in EIP surveillance because they represent a positive CIDT result that is not part of current case definitions. §Data from http://www.cdc.gov/mmwr/preview/mmwrhtml/mm6117a3.htm. ¶Includes confirmed cases only. #Data from http://journals.cambridge.org/abstract_S0195941700093735.  **E. coli, K. pneumoniae, K. oxytoca, E. cloacae, and E. aerogenes nnot susceptible to doripenem, meropenem or imipenem and resistant to all third-generation cephalosporins; *A. baumannii* not susceptible to doripenem, meropenem, or imipenem. ††In 2003, 12% of influenza cases were identified by culture; whereas in 2013, <2% were identified by culture. ‡‡Most samples tested by ABTs at clinical laboratories are sent to public health laboratories for results confirmation by PCR.

Culture remains the mainstay for diagnosis of invasive bacterial and fungal infections that cause predominantly bloodstream infections and meningitis, which are covered under EIP Active Bacterial Core surveillance (ABCs) and Healthcare-Associated Infections Community Interface programs (Table 1). For these pathogens, fulfillment of the surveillance case definition still requires their isolation from a sterile site. Some FDA-cleared multiplex molecular tests for bacterial and fungal bloodstream pathogens are not truly culture independent because they require a positive blood culture from which an organism is identified by PCR (*5**,**6*)*.* In 2014, ≈10% of laboratories that participate in ABCs used one of these platforms to identify species from positive blood cultures (CDC, unpub. data). There are no FDA-cleared molecular tests for directly detecting bacteria from sterile site specimens (e.g., whole blood, cerebrospinal fluid [CSF]), but there are molecular LDTs that are used to directly detect bacterial pathogens from sterile sites. Less than 1% of ABCs laboratories offer these tests for at least 1 of the ABCs pathogens (CDC, unpub. data). There is an FDA-cleared molecular test to detect *Candida* spp. directly from whole blood (*7*)*,* but this test does not seem to be widely used by clinical laboratories (CDC Mycotic Diseases Laboratory, pers. comm.). Nonetheless, multiplex PCR-based tests that detect organisms directly from blood and CSF are under development and may soon become more widely available in clinical settings (*8**,**9*).

For most pathogens covered under the surveillance system for foodborne pathogens (FoodNet, http://www.cdc.gov/foodnet/index.html), culture remains the primary means of diagnosis, but this predominance is changing (*10**,**11*). Antigen-based tests and molecular tests for *Campylobacter* and Shiga toxin–producing *Escherichia coli* have been increasingly adopted by EIP laboratories. Adding positive reports from CIDTs for *Campylobacter* and Shiga-producing *E. coli* results that are not culture confirmed could add an additional ≈13% and ≈8% cases to FoodNet surveillance, respectively (Table 1). FDA recently cleared several molecular enteric syndrome panels (*12*), which are being rapidly adopted (*13*)*.*

## Measuring Disease Burden Trends and Evaluating Public Health Interventions

To assess trends and the effect of population-based interventions over time, methods for measuring disease burden should remain relatively stable or adjustments should be made to account for changes in the use of diagnostic tests. The stability of disease burden estimates will be affected by differences in the performance characteristics of tests used, changes in clinical testing practices, and changes to case definitions. 

### Performance Characteristics and Use of Diagnostic Tests

Accurate tests give positive results when infection is present (i.e., the tests are sensitive) and negative results when infection is absent (i.e., the tests are specific). The predictive value of tests depends, in part, on the prevalence of infection in the population and on whether the organism may be present in the absence of disease (i.e., colonizing body sites). Molecular tests for influenza viruses, *C. difficile*, and *B. pertussis* have been found to be highly sensitive in clinical settings (*14**–**16*). The sensitivity of molecular tests for bacteria may be better than that for culture, particularly when antimicrobial drugs have been administered before specimen collection (*17**–**19*). Highly sensitive molecular tests may produce false-positive results, however, as has been shown in pseudo outbreaks of *B. pertussis* (*20*)*.* Molecular mutations in the organism may result in decreased sensitivity for antigen-based tests (*21*) and molecular tests (*22*). The specificity of CIDTs may be lower than that of culture because molecular targets may be nonspecific for the species of interest (*23*). The influenza and *C. difficile* infection surveillance systems collect data on test method used and adjust national disease estimates on the basis of the sensitivity of the different test types (*3**,**24*).

The availability of tests; their speed, cost, and ease of use; and other factors (e.g., changes in testing guidelines) may result in changes to clinical testing practices, which may affect disease burden trends. These changes may especially be true for pathogens detected by multiplex panels for which clinical suspicion for an organism does not have to be as high as that for a specific organism. If more persons are tested for multiple organisms, more pathogens might be detected. To account for these potential changes, EIP influenza surveillance periodically collects data on the proportion of patients who are tested for influenza if they have an influenza-like illness and adjusts disease burden estimates on the basis of this information (*25*).

### Case Definitions

The case definitions for EIP pathogens include specific requirements for the laboratory methods used and, for some pathogens, the site from which specimens were obtained (Table 1). One consideration is whether clinical symptoms should be included in case definitions because detection of an organism may indicate asymptomatic carriage and not true disease (*26*). This consideration may especially be relevant for organisms that are detected after sample collection from nonsterile sites and that are known to colonize body sites. In EIP, the only activity that includes clinical symptoms as part of the surveillance case definition is Enhanced Pertussis Surveillance. Another consideration may be collection of specimens from negative controls to determine the likelihood of true infection.

In general, EIP case definitions have been characterized by high specificity and high positive clinical predictive value because most rely on culture from a normally sterile body site. Culturing of samples collected from nonsterile sites (e.g., stool samples) may also be more specific than testing for molecular material. EIP decisions about when and how to change case definitions will probably be specific for each pathogen. Advances in the quality of PCR diagnostics led the Council of State and Territorial Epidemiologists to include validated PCR results obtained from sterile site specimens in the Nationally Notifiable Disease Surveillance System for *Haemophilus influenzae* (http://c.ymcdn.com/sites/www.cste.org/resource/resmgr/2014PS/14_ID_05.pdf) and *Neisseria meningitidis* starting in 2015 (http://c.ymcdn.com/sites/www.cste.org/resource/resmgr/2014PS/14_ID_06.pdf). Similarly, campylobacteriosis became nationally notifiable in 2015, and detection of *Campylobacter* spp. by use of any CIDT would be classified as a “probable” case (http://c.ymcdn.com/sites/www.cste.org/resource/resmgr/2014PS/14_ID_09upd.pdf). Like the Council of State and Territorial Epidemiologists, EIP will need to consider what constitutes a valid test. FDA clearance may be a consideration, but FDA-cleared tests may not perform well in real-world clinical settings. LDTs may undergo rigorous validation that may justify including results from those tests. Presenting incidence data stratified by laboratory method (culture-confirmed and positive CIDT reports), as has been done for FoodNet, may be one way to highlight changes to case definitions (*13*).

## Detecting Other Emerging Pathogens

Increased availability and use of CIDTs may increase detection of certain pathogens that were previously hard to identify by culture (particularly those that are part of multiplex panels) or of bacterial pathogens that would otherwise be suppressed by antimicrobial drugs. This increased use may provide the opportunity to conduct surveillance for organisms for which the burden of disease may not have been measured or recognized as emerging infections (e.g., *Mycoplasma pneumoniae,* respiratory syncytial virus, human metapneumovirus, enteroviruses, enterotoxigenic *E. coli*). Additionally, the detection of multiple infectious organisms by multiplex panels could provide insight into polymicrobial interactions and their effect on disease manifestations and severity.

## Analyzing Microbiological and Molecular Characteristics

One of the characteristics that has made EIP surveillance so useful for public health action has been the systematic collection of isolates that enable microbiological and molecular characterization. Serotyping and serogrouping data have been used for developing and evaluating vaccines and for measuring the effectiveness of prevention programs (*27**–**31*). Isolates collected through EIP have been used to identify outbreaks (*32*), monitor and raise awareness of the problem of antimicrobial drug resistance (*33**–**35*), identify mechanisms of resistance (*36*), detect the emergence of new strains (*34*) or mutations that may reduce vaccine effectiveness (*37*), and identify virulence factors (*38*). These isolates have been deposited in national repositories, and streptococcal isolates are widely available to the research communities (http://www.cdc.gov/abcs/pathogens/isolatebank/index.html).

Collection of isolates has been critical for strain characterization by serologic techniques and for in vitro determination of antimicrobial drug susceptibility in EIP reference laboratories. Over time, there has been a shift toward using molecular techniques for strain typing, but both typing and susceptibility testing still rely heavily on the availability of isolates. Although molecular techniques can identify genetic mutations that correlate with phenotypic antimicrobial drug resistance, new mutations may convey the emergence of phenotypes that are not apparent today. Through the CDC Advanced Molecular Detection initiative, EIPs have recently started whole-genome sequencing of EIP isolates (http://www.cdc.gov/amd/project-summaries/emerging-infections.html). Whole-genome sequencing will be used for pathogen characterization for general surveillance and outbreak detection and for exploring genetic determinants of antimicrobial drug resistance, disease severity, and vaccine failure. Some molecular characterization has been performed directly for *N. meningitis* in blood and CSF specimens and for *B. pertussis* in respiratory tract specimens*.* For better characterization of strains without the use of culture, clinical specimens are now collected thorough EIP Enhanced Pertussis Surveillance, as will probably be done for other EIP pathogens in the future. However, much additional research is needed to determine whether and which molecular characteristics can be identified directly from clinical specimens. In the interim, collection of isolates remains essential, as demonstrated by the experience with the *C. difficile* epidemic in the early 2000s, when CIDT use was widespread for this infection and the emergence of the NAP1 strain was not detected until 5 years after steady increases in *C. difficile* incidence and severity (*39*). 

## Future Considerations and Directions 

To continue to impact public health programs and policies, EIPs will have to be forward-thinking in how disease burden trends are measured in light of the continued development and uptake of CIDTs (Table 2). First, EIPs need to continue to systematically monitor the availability and use of these tests through periodic laboratory surveys, either within the EIP network or through coordination with outside organizations and to measure their use in clinical settings. Understanding the use of tests outside of EIP laboratories may also be relevant because some EIPs project estimates of national disease burden. EIPs will also need to develop and regularly evaluate criteria for incorporating CIDTs into case definitions, which will probably vary by pathogen. EIPs can and should contribute to the national discussion about changing case definitions for reportable diseases. Confirmatory testing at public health laboratories may also be necessary for pathogens detected by CIDTs that have questionable performance in real-world settings; however, doing so would require additional public health resources. Performance characteristics need to be determined on an ongoing basis because new variants of organisms that are not detected by the tests may arise. As is already being done for some EIP pathogens, data collection at EIP sites would need to expand to capture information on specific test types and to allow for the reporting of multiple test results. In the era of electronic laboratory reporting, the use of standard test codes that can be transmitted electronically will be essential, and data systems must be able to accommodate more complex data. It will also be critical to perform system checks to avoid counting cases multiple times because >1 testing method may be used for 1 patient. The type of test and the sensitivity and specificity of individual tests and adjustments for changes in testing practices could potentially be incorporated into incidence calculations. After CIDTs have been incorporated into case definitions, EIPs will need to highlight these changes and may consider reporting disease burden by testing method (e.g., cases by culture and molecular testing).

**Table 2:** Plans for measuring disease burden and analyzing microbiological and molecular characteristics in the era of culture independent diagnostics, Emerging Infections Program*

Plan steps
• Conduct periodic laboratory surveys to monitor uptake of tests both within and outside Emerging Infections Program
• Develop and continuously evaluate criteria for accepting CIDTs into surveillance case definitions
• Consider whether results should be confirmed on all or a subset of detections
• Advocate for post-marketing evaluations of CIDTs
• Collect individual test types to account for the sensitivity and specificity of CIDTs
• Adopt methods to account for changes in testing practices that result from use of CIDTs
• Develop an interim strategy for collecting isolates until techniques for serotyping and antimicrobial drug testing on direct patient specimens are available
• Assist in the development and provide specimens to collaborators for the development of microbiological and molecular characterization directly from patient specimens
• Prepare for use of more advanced techniques, like whole-genome sequencing and metagenomics
• Consider performing surveillance for other organisms of public health importance contained in multiplex panels
• Contribute to the understanding of when detection equates with true infection

*CIDT, culture-independent diagnostic test.

Because CIDTs may obviate the need for culture for making a clinical diagnosis, EIPs must consider short- and long-term strategies for assuring the continued availability of isolates. Isolates remain critical for molecular characterization and antimicrobial drug resistance testing. Resources or legal/regulatory approaches may be needed to give clinical or public health laboratories incentives to continue culturing specimens. It is unlikely that clinical laboratories will be paid by insurers for culture in addition to CIDTs. If providing resources to all laboratories is not possible, the EIP network may have a role in providing sentinel sites for collection of isolates. EIP may also have a role in the development and validation of culture-independent methods for serotyping, subtyping, virulence profiling, and antimicrobial drug resistance testing. EIPs have started using banked isolates for developing whole-genome sequence libraries, which will better characterize pathogens at the molecular level and may make characterization from patient specimens (e.g., whole blood, CSF) easier. In the clinical diagnostic setting, metagenomics (the study of genomes from mixed communities of organisms) may eventually replace organism identification, virulence profiling, and some resistance testing, and it may be possible to use this data stream for a variety of public health purposes, including surveillance. Although whole-genome sequencing and metagenomics hold great promise for characterizing pathogens for surveillance, outbreak detection, and detection of emergence of new pathogens, they also pose challenges for processing, analyzing, and interpreting large amounts of data. Resources are needed to develop and sustain the bioinformatics infrastructure and to make sequences available to genomics reference banks so that EIPs can play a broader role in advancing public health practice.

Perhaps the most challenges and opportunities for surveillance systems are presented by use of multiplex tests. They may enable better tracking organisms that are currently underrecognized because culturing is difficult or because they would not otherwise be considered in the differential diagnosis. It may also enable better tracking of polymicrobial infections. However, understanding when detection equates with true infection is a challenge. The EIP may play a unique role in helping to decipher true infections from mere detection of organisms and in describing true polymicrobial infections because laboratory results can be matched with epidemiologic and clinical data.

## Conclusions

The availability and use of CIDTs in clinical medicine present opportunities to rapidly characterize diseases currently covered under the EIP surveillance umbrella and to detect and monitor other emerging infectious diseases. Their use also presents challenges for maintaining the EIP ability to accurately describe disease burdens, the effect of interventions, and microbiological and molecular characteristics of pathogens over time. Because of the long-standing collaboration between the EIP, laboratories, and disease reporters and resources devoted to collecting highly detailed and comprehensive surveillance data, the EIP infrastructure lends itself to close examination of the effect of CIDTs. EIP hopes to work with other domestic and international public health entities, regulatory bodies, diagnostic manufacturers, and academic and clinical groups to chart an evidence-based course for continuing to incorporate CIDTs into public health surveillance.
